# Comparative analysis of functional diversity of rumen microbiome in bison and beef heifers

**DOI:** 10.1128/aem.01320-23

**Published:** 2023-12-06

**Authors:** Thi Truc Minh Nguyen, Ajay Kumar Badhan, Ian D. Reid, Gabriel Ribeiro, Robert Gruninger, Adrian Tsang, Le Luo Guan, Tim McAllister

**Affiliations:** 1 Centre for Structural and Functional Genomics, Concordia University, Montreal, Quebec, Canada; 2 Lethbridge Research and Development Centre, Lethbridge, Alberta, Canada; 3 Department of Animal and Poultry Science, College of Agriculture and Bioresource, University of Saskatchewan, Saskatoon, Saskatchewan, Canada; 4 Department of Agricultural, Food and Nutritional Science, University of Alberta, Edmonton, Alberta, Canada; INRS Armand-Frappier Sante Biotechnologie Research Centre, Laval, Quebec, Canada

**Keywords:** bison, cattle, microbiome, metatranscriptome, rumen

## Abstract

Enhancing crop residue digestibility can boost the available energy for growth and milk yield and improve the sustainability of ruminant production systems. As the natural diet of bison is high in lignocellulose, bison have developed a microbiome that efficiently digests cellulose and hemicellulose. This study compared the rumen metatranscriptomes of bison and beef heifers and investigated the effects of inoculating heifers with bison rumen contents. Gene Ontology, molecular function, and Kyoto Encyclopedia of Genes and Genomes orthology terms identified through gene set enrichment analysis successfully captured differences in gene expression of rumen microbiome between heifers and bison fed different diets. Specifically, differences in nitrogen metabolism was detected between heifers and bison rumen microbiomes. Heifer rumen microbiomes demonstrated a higher dissimilatory nitrate reduction, while bison microbiomes tended to suppress this pathway. In contrast, bison microbiomes expressed higher levels of glutamate dehydrogenase 2 (GDH2) genes. However, glutamate dehydrogenase gene expression was observed to be strictly regulated in heifers, as inoculation with bison rumen contents had no effect on expression. Moreover, the transfer of bison rumen contents led to a persistent downregulation of microbial nitrogen metabolism in heifers after 27 days of transfer. In addition, gene set enrichment analysis also identified the crucial regulatory role of serine/threonine kinase in heifer rumen microbial metabolism and differences in ion transport between heifers and bison. These findings provide valuable insights into the complex interplay between diet and the rumen microbiome.

**IMPORTANCE:**

Ruminants play a key role in the conversion of cellulolytic plant material into high-quality meat and milk protein for humans. The rumen microbiome is the driver of this conversion, yet there is little information on how gene expression within the microbiome impacts the efficiency of this conversion process. The current study investigates gene expression in the rumen microbiome of beef heifers and bison and how transplantation of ruminal contents from bison to heifers alters gene expression. Understanding interactions between the host and the rumen microbiome is the key to developing informed approaches to rumen programming that will enhance production efficiency in ruminants.

## INTRODUCTION

As the world’s population continues to grow and societies become more affluent, the global demand for food, meat, and milk is expected to exponentially increase (1). Meeting this demand in a sustainable manner, with a low environmental footprint is a significant challenge facing humanity. While ruminants are uniquely positioned to satisfy this growing demand by producing high-quality meat and milk protein from forages, crop residues, and food by-products, it is important to note that less than half of the energy in low-quality forages is digestible by ruminants (2). Improving the digestibility of crop residues, such as cereal straw, could provide additional dietary energy to enhance the sustainability of ruminant production systems.

Efforts to introduce pure fibrolytic bacterial cultures into the ruminal microbiome to enhance fiber degradation have largely failed due to a subsequent decline in fitness following culture in the laboratory (3–5). Microbial transplantation has been suggested as a potential method for enhancing the performance of ruminants by reshaping their microbiota (6, 7). Ruminants have benefited from rumen fluid transplantation, a technique that transfers microorganisms from healthy donor animals to recipients, an approach that has been used to treat rumen function disorders (8) associated with ruminal acidosis (9), and displaced abomasum (10).

Non-domesticated ruminants, such as deer, elk, and bison, have distinct rumen microbial communities compared to their domesticated counterparts (11). It has been suggested that the diversity in the composition of feed encountered by wild ruminants contributes to the differences in the gut community observed between wild and domesticated ruminants (11). Bison have a smaller rumen volume, faster liquid dilution rate, and liquid turnover time than cattle, and are superior at digesting fiber (12–14). These differences in microbial and physiological traits may account for the apparent superior ability of bison to digest low-quality forages (15).

In a previous study, we performed inter-species transfer of bison (*Bison bison*) rumen contents to recipient Angus × Hereford cross beef heifers and employed a metataxonomic approach to investigate changes in the rumen microbiota and metabolism. Our findings indicated that the transfer altered the microbial community and enhanced nitrogen digestibility in the recipient heifers (15). This study suggested that inter-species transfer of rumen contents modifies the rumen microbiota, although components of the microbial community did tend to revert back to the pre-inoculation state. All previous efforts to examine the impacts of rumen transfer have used metataxonomic approaches and focus primarily on the bacterial community (16–18). These methods do not reveal information on aspects of gene expression within the microbial community that occur after a major disruption.

In this study, we aimed to investigate the molecular mechanisms underlying functional differences in active microbial metabolism between the domesticated bovine rumen inoculated with rumen contents from captive bison fed a barley silage-oat diet. Specifically, we focus on the impact of inoculating the bovine rumen with bison ruminal contents and assess implications for gene expression and metabolism of the active microbiome. To accomplish this, we performed differential gene expression analysis of the metatranscriptome using RNA from rumen samples collected from bison, heifers, and recipient heifers after day 27 of transfer. Subsequently, gene enrichment analysis was applied to identify major biological processes, molecular functions (MFs), and pathways that exhibit differential expression between bison and heifers. By determining the taxonomic affiliation of these genes, we identify metabolically active phylotypes involved in differential expression. However, we were unable to resolve the effect of time from the effect of transfer, as the experimental design lacked control heifers that did not receive bison contents. Furthermore, diet may have also influenced the results as the diet of bison differed from that of heifers.

## RESULTS

### RNA sequencing, mapping, and taxonomic classification of contigs

A total of 854 M single-end reads were generated as result of sequencing RNA from 36 rumen samples collected from cattle day 0 (*n* = 16), cattle day 27 after rumen transfer (*n* = 16), and bison (*n* = 4) (Table S1a). The contigs from the three different samples were pooled and clustered at 99% similarity, first by their nucleotide sequences and then by the amino acid sequences of their predicted proteins. This yielded 2,768,554 consensus contigs encoding 2,936,003 proteins. Kraken2 (19) was used to map the 2,768,554 assembled contigs. A total of 1,706,811 (61%) contigs matched to nine distinct data sets (Table S1b); among these, a total of 815,293 (29.4%) contigs mapped to the bacterial, archaeal, and protozoan databases (20) combined with the Hungate1000 database (21) and the genomes of *Neocallimastigomycetes* deposited to MycoCosm (22). Interestingly 47.04% (i.e., 1,302,231) of contigs mapped to metagenome-assembled genomes (MAGs) (4,933 MAGS from 240 Scottish heifer’s rumen (23) and 810,108 (29%) contigs successfully mapped to MAG data set consisting of 913 cow rumen microbial genomes (24). Genomic encyclopedia of Bacteria and Archaea (25) mapped 220,078 contigs, while 11,052, 110,436, 2,070, and 1,079 contigs, respectively, were mapped to RefSeq complete fungal, RefSeq complete plant, RefSeq complete viral genomes, and human genomes. Contigs that mapped to plant and human genomes were removed from further analysis. Around 28.15% (779,240) contigs (Table S1b) were mapped to the National Center for Biotechnology Information (NCBI) non-redundant nucleotide database (26).

The predominant taxonomic composition of the rumen samples from bison and the beef heifers is shown in Fig. S1. The taxonomically classified rumen microbiome in this study was composed of ~79% bacteria, ~15% Eukaryota, and ~3% archaea while the remainder were assigned to viruses and unclassified entries. The most abundant bacterial phyla were *Bacillota* formally known as *Firmicutes* (51%), *Bacteroidota* (*Bacteroidetes*, 30%), *Fibrobacterota* (*Fibrobacteres*, 6%), *Spirochaetota* (*Spirochaetes,* 5%), and *Pseudomonadota* (*Proteobacteria*, 2%), while *Ruminococcus* (17%), *Fibrobacter* (12%), *Butyrivibrio* (2%), and *Prevotella* (2%) were the dominant bacterial genera. Contigs associated with eukaryotes accounted for 15% of total classified contigs. Approximately 59% of the eukaryotic contigs were of metazoan or plant origin and were removed from further analyses. Among the remaining eukaryotic contigs, *Neocallimastigomycetes* was found to be the most abundant phylum within the samples. A total of 3% of the contigs were classified as Archaea (*Euryarchaeota*), with *Methanobrevibacter* being the dominant genus (84%), while *Methanomicrobium* and *Methanosphaera* accounted for 8% and 2%, respectively, of *Euryarchaeota* contigs.

### Functional prediction and classification

All proteins predicted from the contigs (2,936,003) were searched using DIAMOND (27) against the annotated protein databases UniProt, JGI rumen protein databases (28), CAZy database (29), and all 16 published rumen protein data sets from the Integrated Microbial Genome (IMG) genome database system (30) (Table 1) and all proteins from metagenomics projects in NCBI (Env_nr) and NCBI-COG2020. Approximately 78% of the proteins showed sequence identity of ≥45% and query coverage of ≥70%, and 59.7% of the proteins had functional assignments (Table S2). However, with a more stringent filter (i.e., %identity ≥95% and query coverage ≥95%), only 18.1% of all proteins identified analogs with ~82% of the proteins being defined as novel. Proteins having a sequence identity and query coverage below 45% and 70%, respectively, were removed from further analysis. Around 46% of the proteins subjected to Gene Ontology (GO) annotation found at least one GO term, while 26% and 37% had a biological process (GO_BP) or molecular function (GO_MF) GO term, respectively. Likewise, 41% of the proteins were assigned to the Kyoto Encyclopedia of Genes and Genomes (KEGG) orthology (KO) group (Table S2).

**TABLE 1:** List of the published rumen protein data sets from the IMG genome database system

IMG genome ID	Genome source	Study name
3300003523	Camel rumen	Camel rumen microbial communities from Jandagh-Isfahan, Iran
2088090000	*Rangifer tarandus platyrhynchus* rumen	*Rangifer tarandus platyrhynchus* rumen microbial communities from Svalbard, Norway
3300020035	Moose rumen	Rumen microbial communities from healthy moose, Palmer, Alaska
3300020024	Moose rumen	Rumen microbial communities from healthy moose, Palmer, Alaska
3300011002	Moose rumen	Rumen microbial communities from healthy moose, Palmer, Alaska
3300020011	Moose rumen	Rumen microbial communities from healthy moose, Palmer, Alaska
3300011005	Moose rumen	Rumen microbial communities from healthy moose, Palmer, Alaska
3300020039	Moose rumen	Rumen microbial communities from healthy moose, Palmer, Alaska
3300011006	Moose rumen	Rumen microbial communities from healthy moose, Palmer, Alaska
3300002597	Camel rumen	Camel rumen microbial communities from Jandagh-Isfahan, Iran
2081372005	*Rangifer tarandus platyrhynchus* rumen	*Rangifer tarandus platyrhynchus* rumen microbial communities from Svalbard, Norway
3300005702	*Rangifer tarandus platyrhynchus* rumen	*Rangifer tarandus platyrhynchus* rumen microbial communities from Svalbard, Norway
3300011008	Moose rumen	Rumen microbial communities from healthy moose, Palmer, Alaska
3300010998	Moose rumen	Rumen microbial communities from healthy moose, Palmer, Alaska
2061766007	Bovine rumen	Switchgrass-associated bovine rumen microbial communities from Urbana, Illinois, USA
2049941000	Bovine rumen	Switchgrass-associated bovine rumen microbial communities from Urbana, Illinois, USA

### Differential expression analysis

Around 587,301 transcripts were differentially expressed between bison and heifer rumen contents (Table S3), and ~39% of them found GO_MF, 28% were assigned to GO_BP while 46% had a KO term. Similarly, ~35%, 25%, and 41% of the differentially expressed proteins (283,855) in day 0 and day 27 rumen samples were assigned to GO_MF, GO_BP term, and KO groups, respectively. Only ~4% of the differentially expressed contigs in different sample types were annotated as CAZy domains.

**Fig 1:**
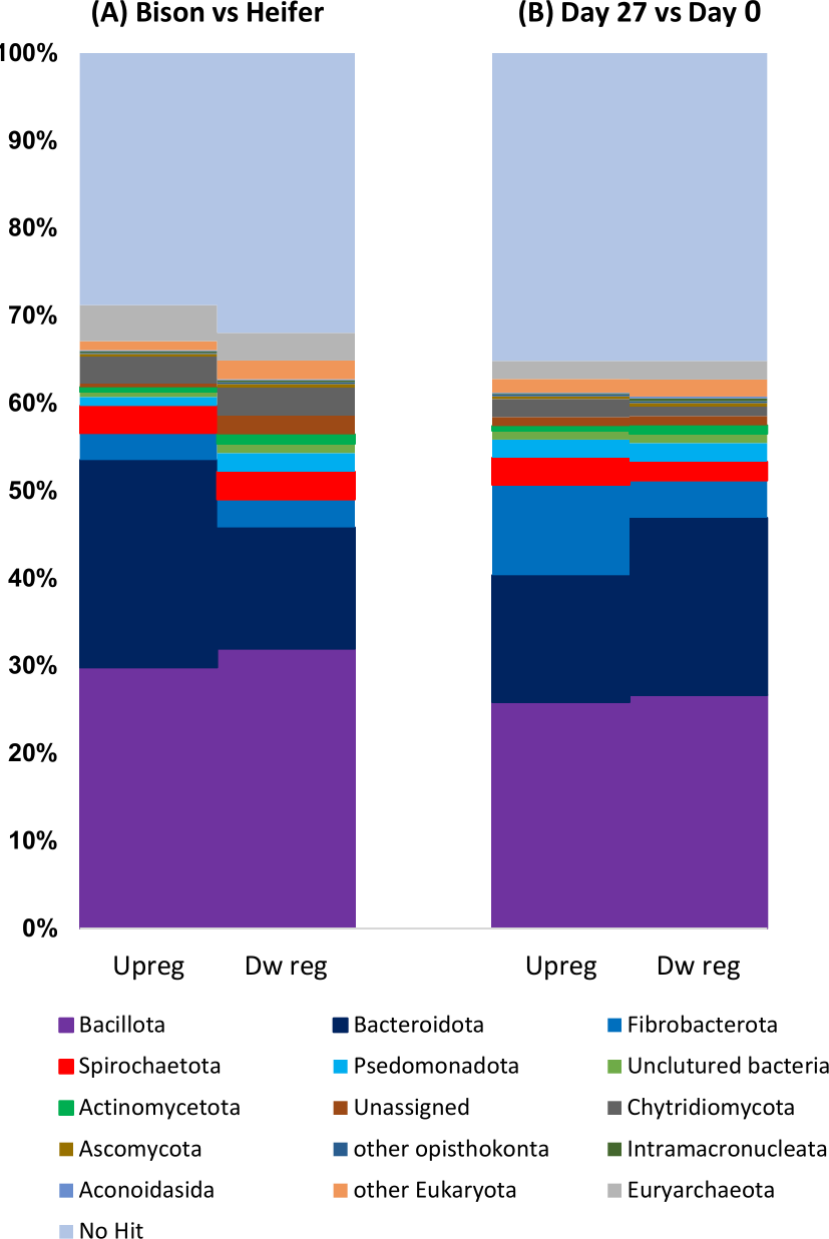
Taxonomic affiliation of differentially expressed transcripts (**A**) in the rumen contents from bison and beef heifers. (**B**) At day 0 and 27 days after transfer of bison rumen contents to heifers.

In the comparisons between bison and heifers, as well as day 0 and day 27, the taxonomic classification of contigs that showed differential expression indicated that 30% of the transcripts did not have any matches in the database (Fig. 1). Among the upregulated contigs in bison, a significantly higher proportion (23%) were affiliated with the phylum *Bacteroidota*, whereas the distribution of differentially expressed contigs associated with other phyla was similar between bison and heifer rumen contents. In contrast, the phylum *Fibrobacterota* had a higher representation in upregulated contigs 27 days after transfer (contributing to 10% of the total differentially expressed contigs, compared to 4% of the downregulated contigs). Furthermore, the downregulated contigs 27 days after transfer had 19% belonging to the phylum *Bacteroidota*, whereas only 14% on the contigs in this phylum were upregulated.

### Gene set enrichment analysis in bison vs heifer rumen samples

**Fig 2:**
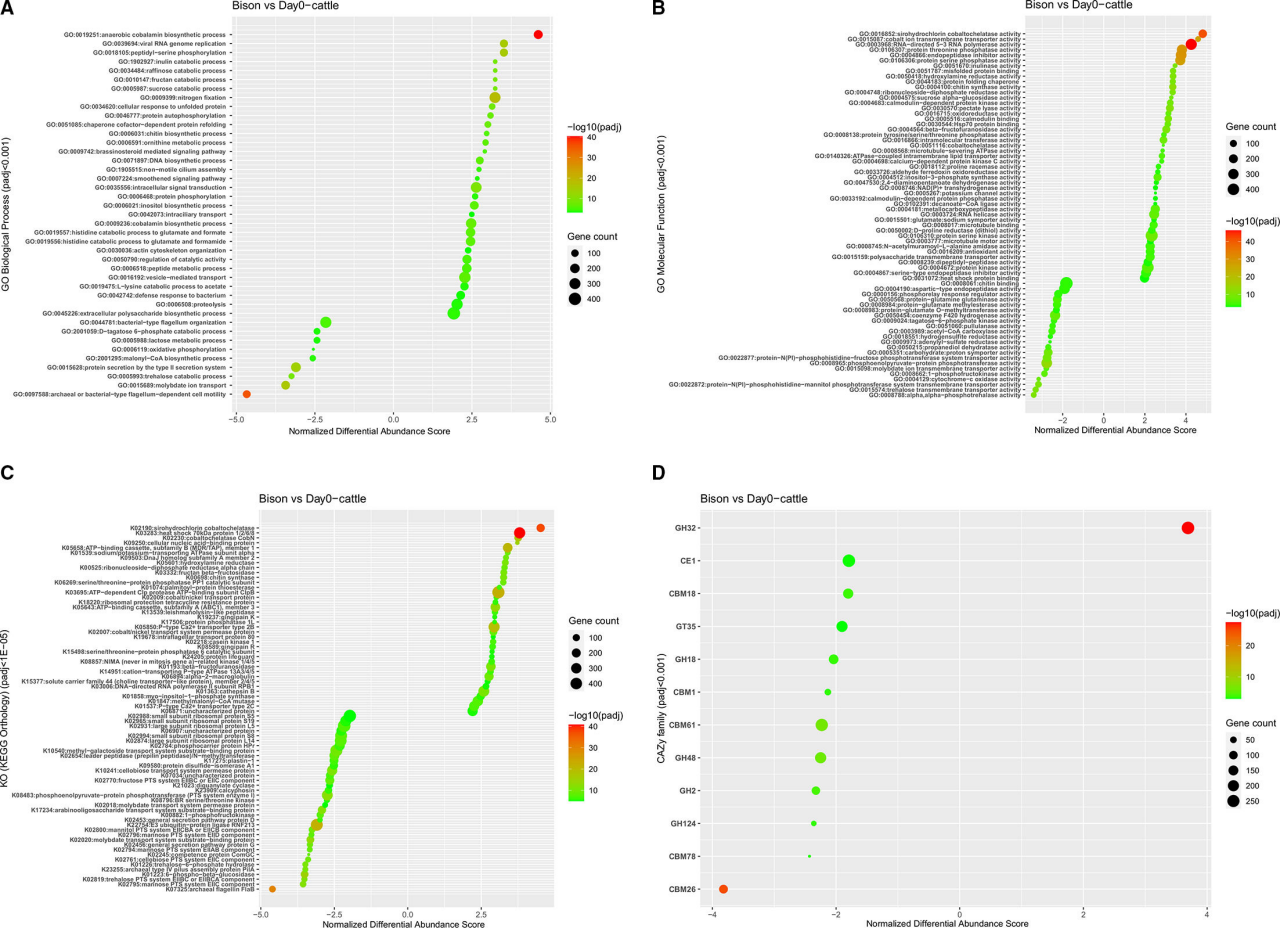
Analyses of functional enrichment of bison vs day 0 (heifer). Bubble graph showing GO function enrichment. (**A**) Biological process, (**B**) MF. (**C**) KEGG pathway enrichment (color bar at right describes significant *P*_adj_-value, and larger bubbles indicate more genes enriched; padj, adjusted *P*-value). (**D**) Differential expression of carbohydrate-active enzymes in rumen contents from bison and heifers (day 0).

For the bison vs heifer comparison, significantly enriched GO terms [adjusted *P*-value (*P*_adj_) <0.1] include 90 biological processes and 137 molecular functions [Supplementary file 1 (Bison_vs_Day0.DESeq2.GSEA)]. The major biological processes enriched in the bison rumen microbiome included the anaerobic cobalamin biosynthetic process (vitamin B_12_), sucrose catabolic process, fructan catabolic process, raffinose catabolic process, inulin catabolic process, nitrogen assimilation, and catabolic glutamate and formate process (Fig. 2A). The prime molecular functions associated with upregulated genes in bison rumen microbiome included sirohydrochlorin cobaltochelatase activity, cobalt ion transmembrane transporter activity, serine phosphatase, hydroxylamine reductase, sucrose alpha-glucosidase, and inulinase activity (Fig. 2B), while genes enriched in rumen samples from heifers were associated with biological processes (Fig. 2A) like molybdate ion transport, trehalose catabolic processes, lactose metabolic processes, D-tagatose 6-phosphate catabolic processes, and flagellum-dependent cell mobility. Major genes enriched in the rumen microbiome from heifers showed molecular functions including alpha, alpha-phosphotrehalase activity, phosphotransferase systems, pullulanase activity, trehalose transmembrane transporter activity, and molybdate ion transmembrane transporter activity (Fig. 2B). A complete list of GO terms and genes involved is listed in Supplementary File 1 (Bison_vs_Day0.DESeq2.GSEA).

Pathways significantly enriched in bison rumen samples (Fig. 2C) included cobaltochelatase, transcription factors, fructose and mannose metabolism, purine and pyrimidine metabolism, chaperones, folding catalysts, and nitrogen metabolism. However, pathways significantly enriched in the heifer rumen contents (Fig. 2C) primarily comprised the carbohydrate phosphotransferase system, starch and sucrose metabolism, and molybdate transport system. The number of differentially expressed genes involved in these KEGG pathways are presented in Supplementary File 1 (Bison_vs_Day0.DESeq2.GSEA), with the top enriched KEGG pathways shown in Fig. 2C.

### Gene set enrichment analysis after the transfer (day 0 vs day 27 samples)

**Fig 3:**
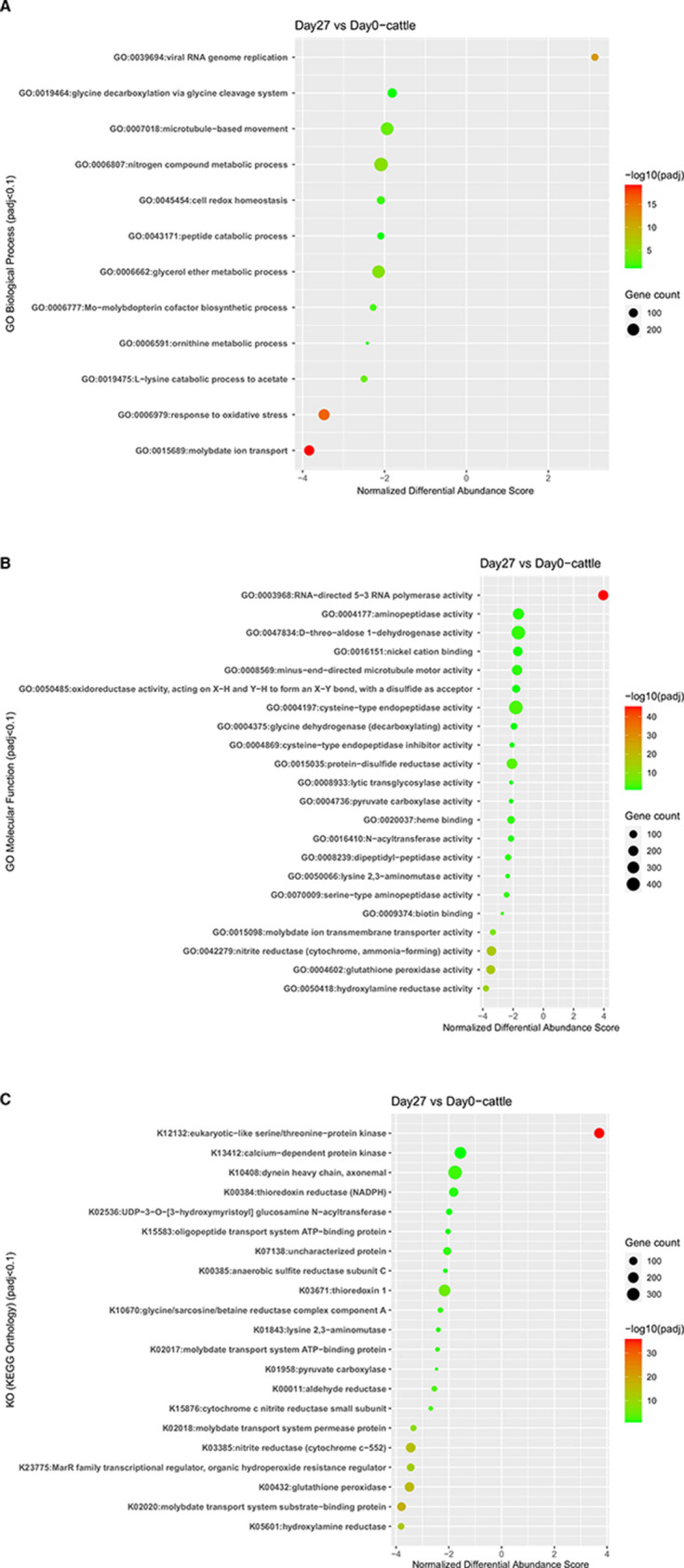
Analyses of functional enrichment 27 days after transfer of bison rumen contents to heifers vs day 0 (heifer). Bubble graph showing GO function enrichment. (A) Biological process, (B) MF. (C) KEGG pathway enrichment (color bar at right describes significant *P*_adj_ value, and larger bubbles indicate more genes enriched; padj, adjusted *P*-value). No significant differential expression of carbohydrate-active enzyme was observed between the day 0 heifer rumen contents and contents collected 27 days after transfer of bison rumen contents to heifers.

A total of 283,855 genes showed differential expression 27 days after transfer of bison rumen contents to heifers (*P*_adj_ <0.1; Fig. 3). A total of 12 biological processes and 22 molecular functions (*P*_adj_ <0.1) were enriched after transfer. Out of the total enriched biological processes, 11 were downregulated after rumen transfer. The major downregulated BP included molybdate ion transport, response to oxidative stress, L-lysine catabolic process to acetate, Mo-molybdopterin co-factor biosynthetic process, and nitrogen compound metabolic process (Fig. 3A). The MFs enriched after transfer were associated with downregulation of hydroxylamine reductase, glutathione peroxidase, nitrite reductase, molybdate ion transmembrane transporter, lysine 2,3-aminomutase, and pyruvate carboxylase activity (Fig. 3B). A complete set of GO terms and differentially expressed genes involved is given in Supplementary File 2 (Day27_vs_Day0.DESeq2.GSEA).

Twenty-two pathways were downregulated in rumen contents collected 27 days after transfer of bison rumen contents to cattle (*P*_adj_ <0.1) [Supplementary file 2 (Day27_vs_Day0.DESeq2.GSEA)], including molybdate transporters, nitrogen metabolism, oxidative stress, lysine degradation, and metabolism of amino acids (Fig. 3C). All KO terms were downregulated following transfer except the KEGG orthology group serine/threonine protein kinase (SPK) (Fig. 3C).

### Taxonomic affiliation of genes within differentially expressed KEGG orthology terms

**Fig 4:**
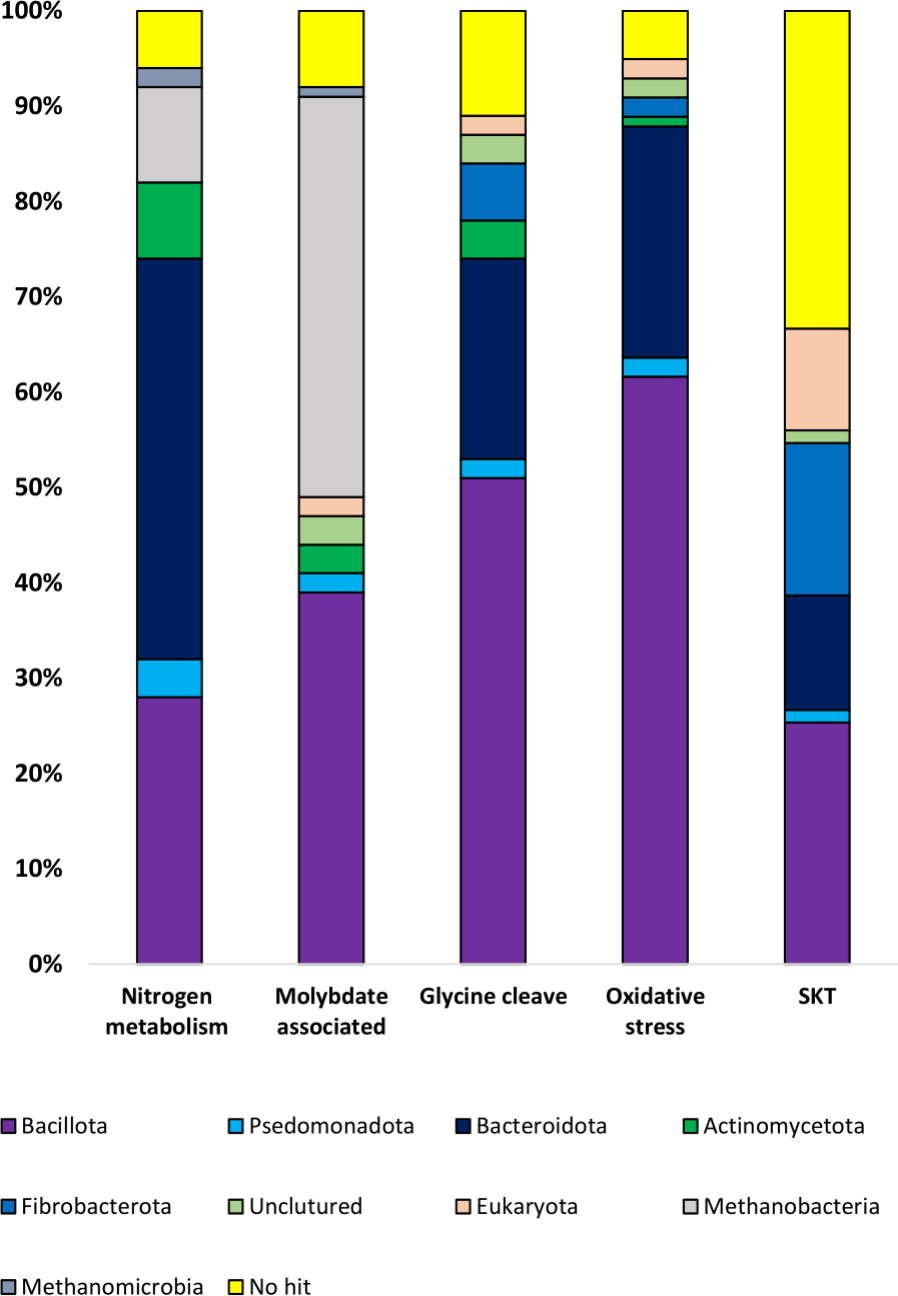
Taxonomic affiliation of differentially expressed KEGG orthology terms associated with downregulated nitrogen metabolism (K03385 , K05601, and K15876, Fig. 3), molybdate transport (K02017, K02020, K02018, Fig. 3), glycine cleavage system (K10670, K01843, K03671, and K00384, Fig. 3), oxidative stress (K23775 and K00432, Fig. 3) and Serine/threonine kinase SKT (K12132, Fig. 3) upregulated orthology terms after transfer.

The taxonomic affiliation of genes associated with identified differentially expressed KEGG orthology was assigned using Kraken2.The molybdate transport system downregulated genes after rumen transfer (K02017, K02020, K02018, Fig. 3) predominantly belonged to *Methanobrevibacter* (42%) (Fig. 4). A significant number of genes within this group were affiliated with *Oscillospiraceae* 17% and uncultured *Eubacteriales* 11% (Fig. 4; Fig. S2A). A total of 83 genes belonging to KO terms associated with nitrogen metabolism (K03385 , K05601, and K15876, Fig. 3) were downregulated after rumen transfer. These genes were predominantly related to the families *Prevotellaceae* (35%), *Oscillospiraceae* (11%), *Lachnospiraceae* (5%), and uncultured *Coribacteriaceae* (7%) (Fig. 4; Fig. S2B), with 7% being affiliated with the methanogenic archaea, *Methanobacteriaceae* (Fig. 4; Fig. S2B). The majority of upregulated phosphotransferase system (PTS) genes in heifers KO (K02770, K08483, K02800, K02796, K02794, K02761, K02819, K02795, Fig. 2) were associated with the *Actinomycetota* (uncultured *Coribacteriaceae,* 23%) and *Bacillota* (*Lachnospiraceae,* 34%) (Fig. 4; Fig. S2C) . Among the glycine cleavage system, KO (K10670, K01843, K03671, and K00384) were downregulated after transfer (Fig. 3), uncultured *Eubacteriales* (17%), *Oscillospiraceae* (11), and *Lachnospiraceae* (10%) were predominant *Bacillota* and uncultured *Bacteroidia* (13%) were the prominent *Bacteroidota* (Fig. 4; Fig. S2D). Similarly, genes as part of KO (K23775 and K00432, oxidative stress) were downregulated after rumen transfer (Fig. 3) and affiliated with the uncultured *Eubacteriales* (18%), *Lachnospiraceae* (17%), and uncultured *Bacteroidia* (Fig. 4; Fig. S2E). Genes associated with the only upregulated KO (SPK) after transfer were affiliated with *Fibrobacter succinogenes* (12%), *Oscillospiraceae* (8%), *Lachnospiraceae* (5%), *Bacteroidia* (3%), and *Prevotellaceae* (3%) (Fig. 4; Fig. S2F).

### Carbohydrate-active enzyme (CAZyme) annotation and taxonomic classification of CAZyme

A total of 79,012 genes (2.69% of the total predicted genes studied in the data set) were annotated as CAZymes (glycoside hydrolase, glycosyltransferase, carbohydrate esterase, polysaccharide lyase, carbohydrate-binding module, auxiliary activity, cohesin, dockerin) using HMMs from the dbCANv9 database (Fig. S3). The majority were annotated as a member of the glycoside hydrolase family (39%) or as genes with carbohydrate-binding modules. A total of 31% of the CAZymes were of unknown function. Around 8.7% of the CAZymes were predicted to be a component of cellulosomes (SLH (S-layer homology)/cohesin/dockerin), while 7% were predicted to be carbohydrate esterases and 11% as glycosyl transferases (Fig. S3). The majority of the CAZyme (~56%) were assigned to bacteria (Fig. S4), while 11% of the CAZyme pool was assigned to the Eukaryota. Dominant bacterial phyla included *Bacillota* (46%), *Bacteroidota* (33%), *Fibrobacterota* (12%), *Spirochaetota* (3%), and *Pseudomonadota* (2%). *Ruminococcus* (17%), *Butyrivibrio* (2%), *Fibrobacter* (12%), and *Prevotella* (2%) were the most abundant genera. Most of the transcripts among Eukaryota were observed to be associated with fungi (81%) and *Alveolata* (9%).

The majority of glycoside hydrolases encoding genes were associated with bacteria (65%) (Fig. S5). *Bacillota* (48%), *Bacteroidota* (30%), and *Fibrobacterota* (12%) were the most dominant phyla found to harbor glycoside hydrolase genes. The most observed bacterial genera were *Ruminococcus* (18%), *Fibrobacter* (12%), *Butyrivibrio* (2%), and *Prevotella* (2%) (Fig. S5). Eukaryotes accounted for ~12% of the glycoside hydrolase pool, with 70% of them associated with fungi. Most of these fungal glycoside hydrolases were from *Neocallimastigomycetes* [*Neocallimastix* (24%)], *Caecomycetes* (27%), *Piromyces* (24%), *Pecoramyces* (10%) and *Anaeromyces* (7%). Approximately, 99% of the glycoside hydrolases from *Alveolata* were assigned to the *Ciliophora* (Fig. S5) with *Epidinium* (46%), *Polyplastron* (24%), and *Eudiplodinium* (14%) being dominant genera. Around 19% of the glycoside hydrolase proteins remained unassigned.

### Differential expression of CAZymes

Metatranscriptomic data were used to evaluate the differential expression of genes encoding specific CAZyme families between the bison and heifer ruminal microbiome. Differential gene expression analysis suggested beef heifers overexpressed cellulases (glycoside hydrolyases : GH 44, GH 48, GH 64, GH 74, and GH 124) and cellulose binding module (CBM) with predicted binding functions to cellulose (CBM 1, CBM 3, CBM 30), xyloglucan (CBM 76, CBM 78, CBM 80), and xylan (CBM13, CBM 22) (Fig. 2D). In contrast, the bison microbiome overexpressed GH 32 (inulinase), an enzyme involved in fructan digestion. However, no significant differential CAZyme expression was noted 27 days after the transfer of bison rumen contents to cattle.

## DISCUSSION

Studies have shown that bison typically graze lower-quality forages and are more efficient than cattle when it comes to fiber digestion and nitrogen metabolism (12, 31, 32). In a previous study, we examined the effects of repeatedly inoculating beef heifers with bison rumen contents on rumen microbiota and fiber digestibility (15). The rumen transfer initially altered the rumen bacterial community in heifers, but it tended to revert to its the original composition 27 days after transfer. Inoculation with bison contents did not increase microbial protein synthesis or fiber digestibility (15).

The enrichment analysis of metatranscriptome data reported here effectively captures the microbial function differences in response to the different diet between bison and heifers. The bison used as donors for inoculation were fed a diet consisting of barley silage and oats (75:25 dry matter [DM] basis), while the recipient heifers were fed a barley straw- and concentrate-based (70:30 forage to concentrate, DM basis) diet. These diets were deliberately formulated in this manner to maximize the opportunity of observing post-inoculation differences. The microbiome of bison was enriched in fructan/sucrose/raffinose/inulin catabolic processes, as well as exhibiting upregulated inulinase expression (Fig. 2). This is consistent with the high fructan content of the oat and barley silage diet. Winter cereals like oat accumulate fructans during a cold hardening period that is frequently preceded by sucrose accumulation (33). In contrast, the diet fed to heifers contained distiller corn dry grain with soluble (DDGS) and canola meal, resulting in enrichment of genes related to trehalose/lactose/D-tagatose catabolic processes, with upregulated pullulanase, alpha, alpha-phosphotrehalase, and trehalose transmembrane transporter activity as result of the soluble carbohydrate and disaccharide content. Active sugar transport and phosphorylation by the microbial community in heifers is reflected by the enrichment of various genes encoding PTS. Likewise, phospho-β-glucosidases that degrade phosphorylated glucosides and fiber-related disaccharides were also enriched in the heifer microbiome compared to bison. This suggests active carbohydrate solubilization from concentrate (corn DDGS) due to limited carbohydrate availability in the diet, which primarily consisted of recalcitrant barley straw.

**Fig 5:**
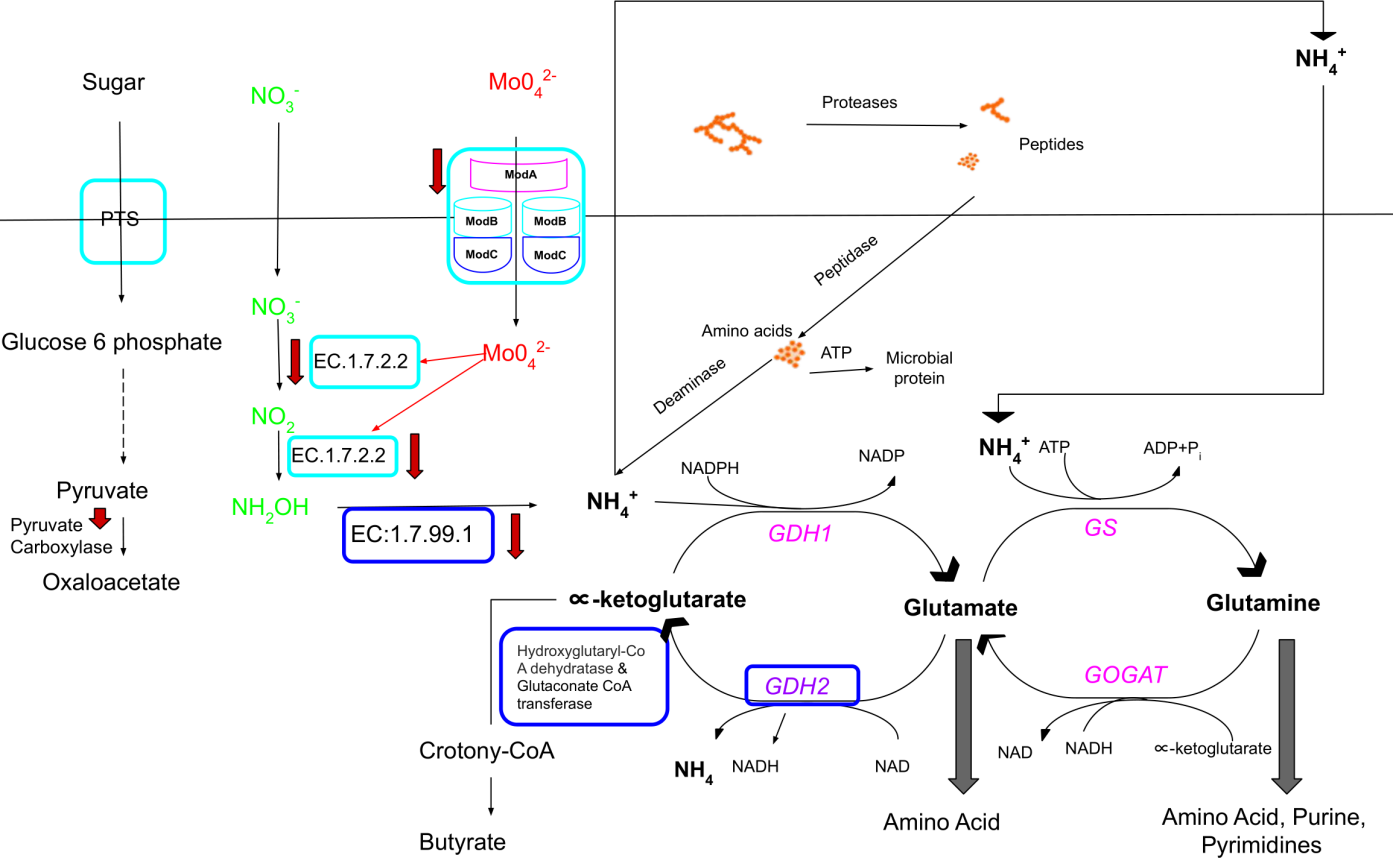
Graphical abstract representing the molecular basis of differences observed between heifer and bison rumen metabolism and the effect of the transfer on microbial metabolism. Cyan box represents molecular functions upregulated in the heifer microbiome. Dark blue box represents molecular functions observed to be upregulated in the bison microbiome. Magenta text represents ammonia assimilatory pathway (glutamate synthase: GS-GOGAT) to nitrogen compounds. Purple (GDH2-bison upregulated) represents catabolic glutamate conversion to α-ketoglutarate. Red down arrows represent the molecular functions downregulated after transfer of bison rumen contents to heifers: nitrate(NO_3_^−^), nitrite (NO_2_), hydroxy-amine (NH_2_OH), molybdate (Mo0_4_^2^), sugar phosphotransferase system PTS, ammonia (NH_4_^+^). EC:1.7.2.2, nitrite reductase (*nrfA* and *nrfH*). EC:1.7.99.1, hydroxylamine reductase (hcp).

Enrichment analysis also identified a difference in nitrogen metabolism between the microbiome of bison and heifers (Fig. 5). In heifers, the microbiome was enriched for genes associated with molybdate ion transport and nitrate reductase *narB* (ferredoxin-nitrate reductase) activity (Fig. 2). Molybdate plays a critical role in several prokaryotic and eukaryotic metabolic pathways including carbon, sulfur, and nitrogen metabolism (34). Molybdate has been reported to play a critical role in nitrate reduction (35), and is required for the reduction of nitrate to ammonia through molybdoenzyme nitrate reductase (36). The transport of molybdate into the bacterial cell is mediated by a high-affinity molybdate transport system comprised of ModA, ModB, and ModC, all of which were downregulated after transfer of bison contents to the rumen of heifers (Fig. 3 and 5). Likewise, nitrite reductases (*nrfA*, *nrfH*) were also downregulated (Fig. 3; Fig. S2) after the transfer. Efficient nitrogen recycling is one strategy that ruminants use to remain productive when fed low-protein diets and the protein requirements of bison would be lower than heifers (31, 32). As a result, it is unlikely that bison benefit from non-protein nitrogen sources such as nitrate, as evidenced by their lower level of expression of genes encoding molybdate transport and nitrate reductase as compared to heifers. It is possible that this adaptation evolved as a response to the fluctuating levels of nitrate present in the natural diet of bison, which can be influenced by factors such as seasonality, plant maturity and plant stressors like drought or disease. Unlike the conversion of nitrate to nitrite, the reduction of nitrite to ammonia is slower in ruminants, leading to the accumulation of nitrite. High levels of nitrite can be toxic, as it binds to hemoglobin and interferes with oxygen transport, forming methemoglobin, a life-threatening condition. The bison microbiome may have adapted to enhance the conversion rate of nitrite to ammonia by significantly enriching hydroxylamine reductase [enzyme commission (EC): 1.7.99.1], while expressing lower levels of nitrate reductase than heifers (Supplementary File 1).

Transfer of bison ruminal contents led to a downregulation of the expression of molybdate transport system genes in heifers, and the majority of these genes were found to be associated with *Methanobacteriaceae* (archaea), *Oscillospiraceae*, and *Eubacteriales* (Bacteria). *Methanobacteriaceae* typically use H_2_ + CO_2_, or H_2_ + CO_2_ and formate as substrates for methanogenesis. The molybdoenzyme methanofuran dehydrogenase plays a crucial role in the initial step of the reduction of CO_2_ to CH_4_. Interestingly, only molybdoenzyme nitrate (*narB*) and nitrite reductase ((*nrfA*, *nrfH*)) showed differential expression due to the disrupted molybdate transport system, with no indication of an impact on the expression of enzymes involved in sulfur metabolism or methanogenesis. Therefore, it is plausible that molybdate was taken up by an alternative transport system. In fact, earlier studies have suggested the potential transport of molybdate via a sulfate transporter (37).

In ruminants, the synchronized supply rate of ruminal energy and nitrogen sources is critical to microbial protein synthesis (38). It is important to note that the barley silage in the donor bison diet was less recalcitrant compared to the barley straw-based diet fed to the beef heifers (39). As a result, the CAZy profile in the ruminal microbiome of heifers exhibited greater expression of genes associated with fibrolytic enzymes than bison. In the heifer microbiome, most overexpressed genes encoding enzymes targeted cellulose and hemicellulose, while the bison ruminal microbiome overexpressed inulinase (GH 32), targeting fructosyl linkages which would have been enriched in the oat-based diet of bison. This observation aligns with the fact that transfer of ruminal contents from bison to cattle did not have an effect on feed intake, average daily gain, or the apparent total digestibility of dry matter, organic matter, neutral detergent fiber, or acid detergent fiber (15).

Prokaryotic GDH typically operates with either NADP+ (EC 1.4.1.4) or NAD+ (EC 1.4.1.2) as co-factors (40). The GDH1 enzyme (EC 1.4.1.4), which is specific to NADP+, is generally responsible for nitrogen assimilation via α-ketoglutarate amination (Fig. 5). Conversely, the expression of GDH2, which produces an enzyme (EC 1.4.1.2) specific to NAD+ was upregulated in bison ruminal contents and would facilitate glutamate catabolism to ammonia and α-ketoglutarate (40) (Fig. 5). GDH1 along with the GS-GOGAT (glutamine synthetase-glutamate synthetase) system converts ammonia into glutamine and glutamate, which serve as nitrogen donors in the formation of cellular nitrogenous compounds (amino acids, purine, and pyrimidines) (Fig. 5). Although the GS-GOGAT (glutamine synthetase- glutamate synthetase) system is a key pathway for ammonia assimilation, it is highly ATP-dependent and can be quickly inactivated in the presence of ammonia (40, 41). There was no significant difference in the expression of the GS-GOGAT system between the bison and heifer ruminal microbiomes. The GDH2 pathway has a low energy cost relative to GDH1 (GS-GOGAT) (40, 42) and was upregulated in the bison ruminal microbiome. Overexpression of *GDH2* in the bison microbiome suggests breakdown of glutamate to produce α-ketoglutarate and ammonia (Fig. 5; Fig. S1). Bison microbiota may have developed an adaptation to surviving on a low-energy, low-protein diets by increasing the expression of GDH2. GDH2 converts glutamate to α-ketoglutarate and ammonia, which balances carbon catabolism with nitrogen metabolism, conserving energy while directing the flow of reducing equivalents toward ATP (43). This adaptation allows the carbon derived from glutamate to be used as a source of fuel for the tricaboxylic acid cycle (TCA cycle). α-Ketoglutarate is a substrate for microbial succinate production that can be converted into propionate and butyrate via crotonyl-CoA. Fermentation pathways convert glutamate to ammonia (Fig. 5), CO_2_, acetate, butyrate, and H_2_ via 2-hydroxyglutarate (44). The bison microbiome overexpressed hydroxyglutaryl-CoA dehydratase and glutaconate CoA-transferase (Supplementary File 1) genes, suggesting an active role for glutamate fermentation via 2-hydroxyglutarate. However, the transfer of bison ruminal contents to beef heifers did not alter GDH expression in microbiota.

The only enriched KO term observed after rumen transfer was serine/threonine kinase (STK). Serine/threonine kinase plays a crucial role in regulating various cellular processes. Previous studies have linked serine/threonine kinases to metabolic regulation, including oxidative stress, nitrogen assimilation, central metabolism, and glutamate catabolism, as well as their role in growth and development in prokaryotes and eukaryotes (45–49). Given the repression of nitrogen assimilation, oxidative stress response, and amino acid metabolism and upregulated expression of serine/threonine kinase after bison rumen content transfer, it is reasonable to assume that serine/threonine kinases played a significant regulatory role in rumen microbial metabolism. Accordingly, the taxonomic classification of genes associated with various downregulated KO (as shown in Fig. S2A through E) revealed that specific taxa, including *Oscillospiraceae*, *Lachnospiraceae*, uncultured *Eubacteriales*, *Prevotellaceae*, *Bacteroidia*, and *Fibrobacter succinogen*es, downregulated microbial metabolism following transfer. Furthermore, upregulated serine/threonine kinases genes were also found to be affiliated with the same core taxa *Oscillospiraceae*, *Lachnospiraceae*, *Bacteroidia*, and *Prevotellaceae* (as depicted in Fig. S2F), indicative of their regulatory role of serine/threonine kinases.

### Conclusion

This study delves into the molecular-level variation in nitrogen metabolism between heifers and bison rumen contents, especially in low dietary energy conditions. The results showed that the ruminal microbiome in beef heifers displayed higher levels of dissimilatory nitrate reduction, whereas the bison rumen microbiome seemed to suppress this pathway. The bison ruminal microbiome had higher expression of GDH2, a critical enzyme in central metabolism that diverts carbon skeletons away from nitrogen metabolism toward energy-generating carbon metabolism and volatile fatty acid synthesis. However, GDH expression was found to be tightly regulated in beef heifers ruminal contents, and bison rumen content inoculation had no effect on its expression, whereas nitrogen metabolism remained downregulated after bison transfer, indicative of a persistent change in microbial nitrogen metabolism in heifers. The study also highlighted the significant regulatory role of STKs in rumen microbial metabolism, which can be an effective tool to modulate rumen microbiome activity. This study highlights crucial role of ruminal microbiome in the adaptation of ruminants to their natural diet and environmental factors, and the need for further research into understanding the functional diversity of microbiomes in different species and interactions between the rumen microbiome and its host.

## MATERIALS AND METHODS

### Rumen transfer and RNA extraction

The details of the bison rumen content transfer experiment have been described previously (15). In brief, the ruminal content transfer was carried out twice to increase the chances of establishment of microbes associated with the donor bison inoculum within the rumen of recipient 2-year-old nonpregnant heifers (15). Whole rumens from bison (*n* = 32) fed a barley silage-oat (75:25) diet were collected at a commercial abattoir immediately after slaughter, sealed, and transported in insulated container in heated truck to a barn. Contents were stored in holding tank maintained as 39°C under a constant flush of O_2_-free CO_2_ (15). The rumen contents of individual ruminally cannulated heifers (*n* = 16, age  = 14  ±  2 months) fed 70:30 barley straw:concentrate diet (15) were evacuated, collected in a sealed container, mixed, and weighed. A 30% (wet wt) portion of the heifer rumen contents was returned back to the respective heifer to maintain barley straw-adapted microbes, while the remaining 70% (wet wt) of the individual heifer contents was replaced by pooled bison rumen contents. The entire transfer experiment was repeated a second time after 2 weeks. Rumen content samples from individual heifers (day 0 heifer) and the pooled bison (bison) were collected immediately before the rumen transfer and before feeding. Likewise, rumen contents from each heifer were collected before the morning feeding, 27 days after the second rumen transfer (day 27). The solid and liquid phases of the collected samples were separated using a bodum coffee filter plunger (Bodum Inc., Triengen, Switzerland). Aliquots of solid digest (~5 g) were immediately flash frozen in liquid nitrogen and stored at −80°C for further processing. The collected solid digesta was ground to a fine powder in liquid nitrogen using a Retsch RM100 grinder (Haan, Germany) and processed for total RNA extraction as described previously (50). The RNA quality was determined using RNA 6000 nano chip (Agilent Technologies, Mississauga, Ontario, Canada) on an Agilent 2100 BioAnalyzer (Agilent Technologies), with RNA integrity number (RIN) values of 7–8 considered acceptable for further analysis.

### Sequencing data pre-processing, contig assembly, and protein prediction

RNA sequencing was conducted on rRNA-depleted library [KAPA rRNA-depleted (bacteria) preparation kit using the HiSeq 4000 PE100]. Reads were cleaned of sequencing adapters using Skewer (51). Ribosomal RNA was removed from the trimmed reads with SortMeRNA (52). The cleaned reads from each of the three read sets were separated and assembled into contigs using Megahit (53). Contigs were oriented in the sense direction by aligning the strand-specific reads to them and, if necessary, splitting contigs to obtain homogeneous orientation. The contig sequences were translated in three frames and the longest predicted peptide without internal stop codons was taken as the predicted protein for the contig. The contigs from the three read sets were pooled and clustered with uclust (54). Contigs were first clustered by their nucleotide sequences and then by their amino acid sequences based on their predicted proteins. The clustering yielded 2,768,554 consensus contigs encoding 2,936,003 proteins, with 5% of the total transcripts encoding multiple proteins. The abundance of each consensus contig in each sample was estimated using Salmon to count the reads that mapped to each specific contig (55). Sequence data are available at NCBI Bioproject # ID: PRJNA1005307.

### Taxonomic assignment of consensus contigs

Kraken2 (19) was used to perform the taxonomic classification of the consensus contigs. Multiple reference databases were built from genomes collected from different sources: complete genomes from RefSeq including from bacteria, archaea, protozoa, fungi, plants, viruses, and humans (20); 12 genomes originated from the *Neocallimastigomycetes* group in MycoCosm database (22); 410 rumen microbial genomes from Hungate1000 Project (21); 913 microbial genomes from cow rumen (24); 4,933 MAGs from 240 Scottish cattle rumen (23); 974 bacterial and 29 archaeal species from the Genomic Encyclopedia of Bacteria and Archaea (25); and NCBI non-redundant nucleotide database (26). For contigs having multiple taxonomic assignments as a result of mapping to different reference databases, the lowest common ancestor was selected for the final assignment using in-house perl scripts. The microbe community identified from Kraken2 was further examined by krona pie charts (56).

### Functional prediction and classification

The function of 2,936,003 proteins encoded by the consensus contigs were predicted by running diamond (27) search against reference databases including UniProt (28), CAZy (29), all 16 published rumen protein data sets from the IMG genome database system (30) (Table 1), and all proteins from COG2020 database (https://ftp.ncbi.nlm.nih.gov/pub/COG/COG2020/data/) and from metagenomics projects (https://ftp.ncbi.nlm.nih.gov/blast/db/) in NCBI. All hits with percent identity below 45% and query coverage less than 70% were discarded. Functional information including protein names, GO terms, enzyme commission (EC) numbers, KO, and Cluster of Orthologous Genes (COG) assignments were transferred from the best diamond hits. Moreover, kofam scan v1.3.0 (57) and GhostKOALA (58) were used to increase the mapping rate of predicted proteins into KO groups. For query proteins having multiple hits from different reference databases, those alignments with the highest percent of identity were selected.

### Identification of CAZymes

The CAZyme domain detection was performed by running hmmscan from HMMER v3.3 package (http://hmmer.org/) against dbCANv9 database (59). Only hits satisfying the cut-off values (if alignment >80 aa, use *e*-value <1e − 5, otherwise use *e*-value <1e − 3; covered fraction of HMM >0.3) were further analyzed.

### Differential gene expression and gene set enrichment analyses

Gene abundance was estimated using Salmon to get the transcript per million values, with differentially expressed genes between samples analyzed using DEseq2 (60) at an adjusted *P*-value (*P*_adj_) below 10%. The gene list from DESeq2’s output was used to determine GO terms, CAZy families, and KO pathways that were significantly enriched. The gene set enrichment analysis was performed by combining results from the two R packages, GAGE (61) and FGSEA (62), with *P*_adj_ <0.1. The significantly enriched pathways were examined by scatter dot plots created using the ggplot2 package (63).

## Data Availability

All sequence data is available at NCBI Bioproject # ID: PRJNA1005307.

## SUPPLEMENTAL MATERIAL

Supplemental file 1 aem.01320-23-s0001.xlsx Bison_vs_Day0.DESeq2.GSEA_noMetazoa_noplants.

Supplemental file 2 aem.01320-23-s0002.xlsx Day27_vs_Day0.DESeq2.GSEA_noMetazoa_noplant.

Supplemental file 3 aem.01320-23-s0003.pdf Fig. S1 to S5 and Tables S1 to S3.

ASM does not own the copyrights to Supplemental Material that may be linked to, or accessed through, an article. The authors have granted ASM a non-exclusive, world-wide license to publish the Supplemental Material files. Please contact the corresponding author directly for reuse.
