# Supplementary material for: Comparative analysis of functional diversity of rumen microbiome in bison and beef heifers
Source: Appl Environ Microbiol. 2023 Dec 6;89(12):e01320-23. doi: 10.1128/aem.01320-23 (PMC10734544; doi:10.1128/aem.01320-23)
Supplement: Supplemental file 3 — Fig. S1 to S5 and Tables S1 to S3. [file aem.01320-23-s0003.pdf]

Supplementary Figure 1: Taxonomic contribution of all contigs (based on contig counts)

Dominant bacterial phyla

- Firmicutes (51%)
- Bacteroidetes (30%)
- Fibrobacteres (6%)
- Spirochaetes (5%)
- Proteobacteria (2%)

Dominant Genus

1. Ruminococcus (17%)
2. Butyrivibrio (2%)
3. Fibrobacter (12%)
4. Prevotella (2%)

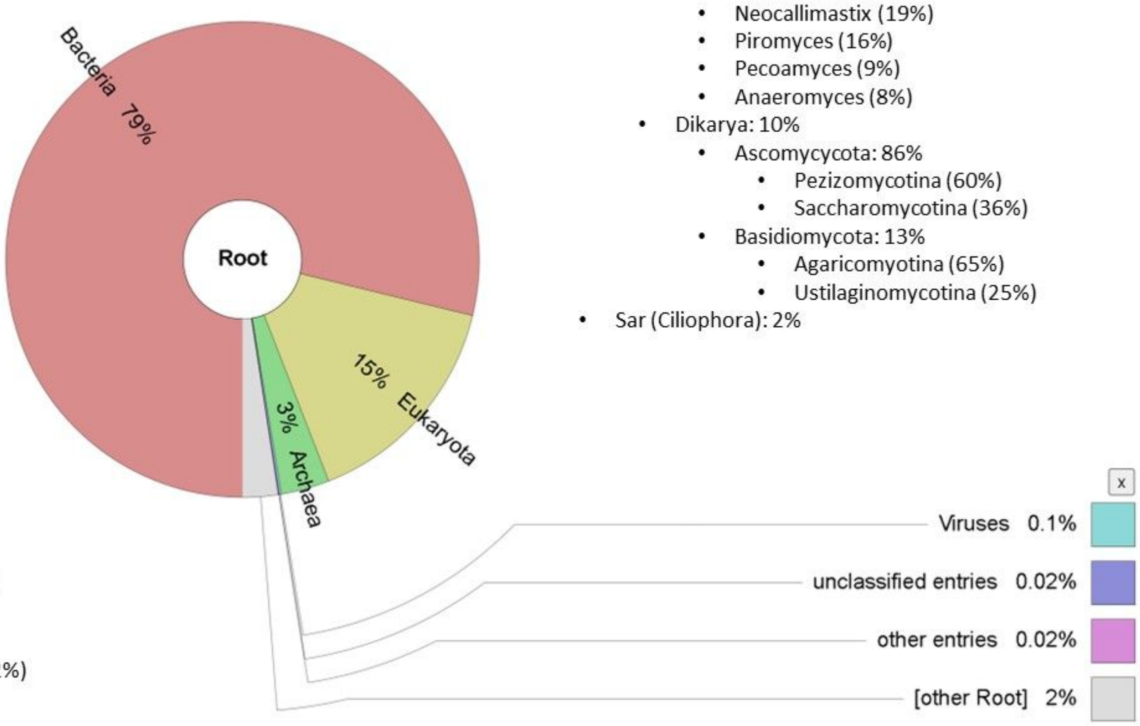

- **Fungi: 27%**
  - Neocallimastigomycetes: 89%
    - Caecomyces (44%)
    - Neocallimastix (19%)
    - Piromyces (16%)
    - Pecoamyces (9%)
    - Anaeromyces (8%)
  - Dikarya: 10%
    - Ascomycycota: 86%
      - Pezizomycotina (60%)
      - Saccharomycotina (36%)
    - Basidiomycota: 13%
      - Agaricomyotina (65%)
      - Ustilaginomycotina (25%)
- Sar (Ciliophora): 2%

- Euryarchaeota (96%)
  - Methanobrevibacter (84%)
  - Methanomicrobium (8%)
  - Methanosphaera (2%)
- Candidatus Thermoplasmatoda (2%)

Supplementary figure 2: Taxonomic affiliation of genes belonging to KO terms associated (A) Molybdate transport (K02017, K02020, K02018, Figure 3), (B) nitrogen metabolism (K03385 , K05601, and K15876, Figure 3) (C) PTS (K02770, K08483, K02800, K02796, K02794, K02761, K02819, K02795, Figure 2) , (D) Glycine cleavage system (K10670, K01843, K03671, and K00384, Figure 3) (E) Oxidative stress (K23775, and K00432, Figure 3) were downregulated and (F) SKT (K12132, Figure 3) upregulated after transfer.

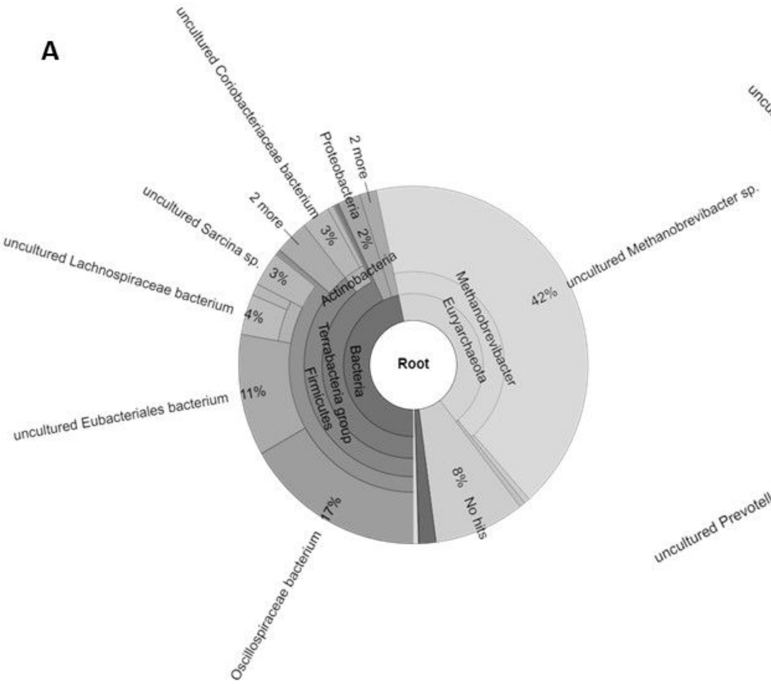

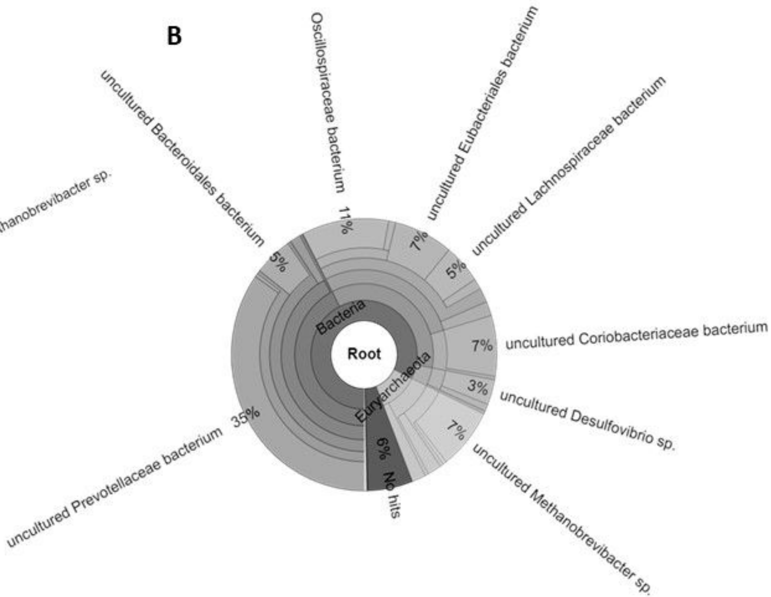

C

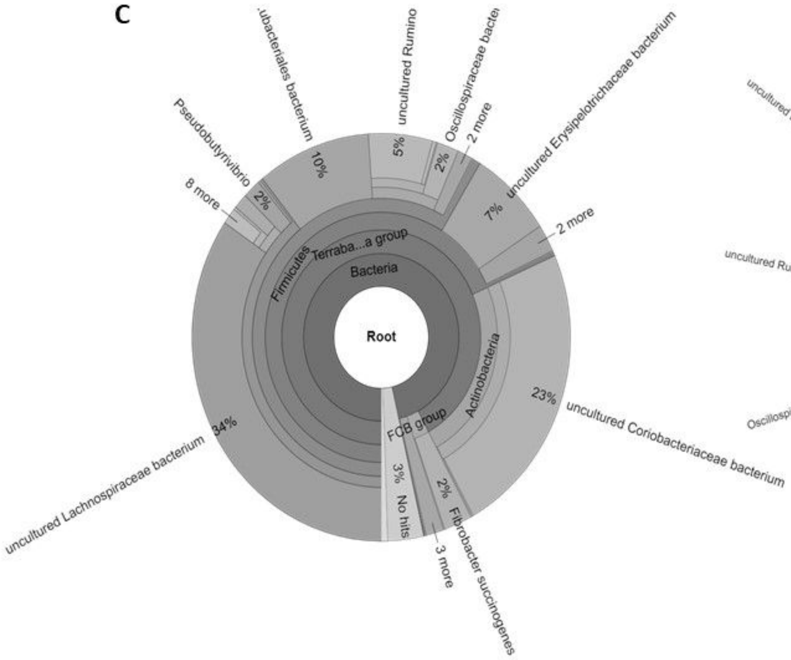
Root
Bacteria
Terraba...a group
Firmicutes
Actinobacteria
FCB group
No hits
3%
34%
uncultured Lachnospiraceae bacterium
23%
uncultured Coriobacteriaceae bacterium
10%
ubacteriales bacterium
5%
uncultured Rumino
2%
Oscillospiraceae bacte
2 more
7%
uncultured Erysipelotrichaceae bacterium
2 more
Pseudobutyrivibrio
2%
8 more
2%
Fibrobacter succinogenes
3 more

D

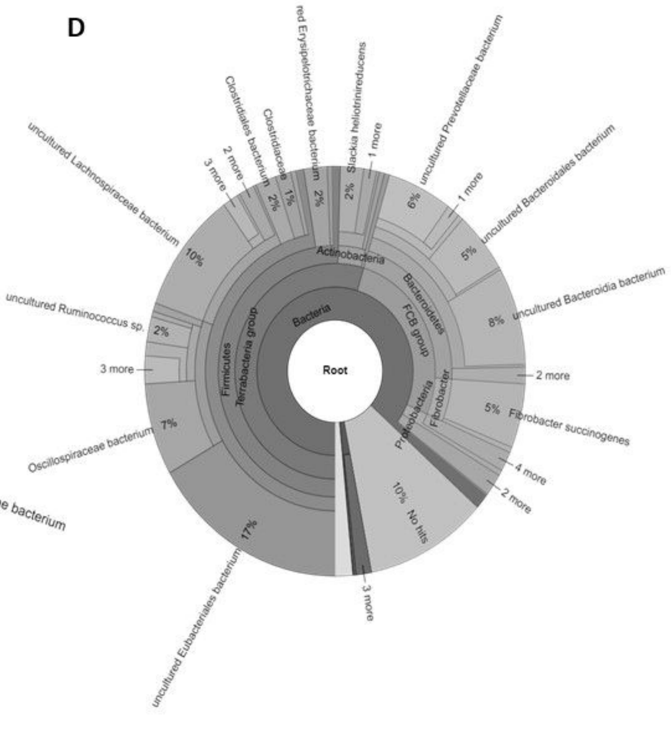
Root
Bacteria
Terrabacteria group
Firmicutes
Actinobacteria
Bacteroidetes
FCB group
Proteobacteria
Fibrobacter
10%
No hits
17%
uncultured Eubacteriales bacterium
10%
uncultured Lachnospiraceae bacterium
7%
Oscillospiraceae bacterium
2%
uncultured Ruminococcus sp.
3 more
3 more
2 more
Clostridiales bacterium
2%
Clostridiaceae
1%
red Erysipelotrichaceae bacterium
2%
Slackia heliotrinireducens
2%
1 more
6%
uncultured Prevotellaceae bacterium
1 more
5%
uncultured Bacteroidales bacterium
8%
uncultured Bacteroidia bacterium
2 more
5%
Fibrobacter succinogenes
4 more
2 more
3 more

E

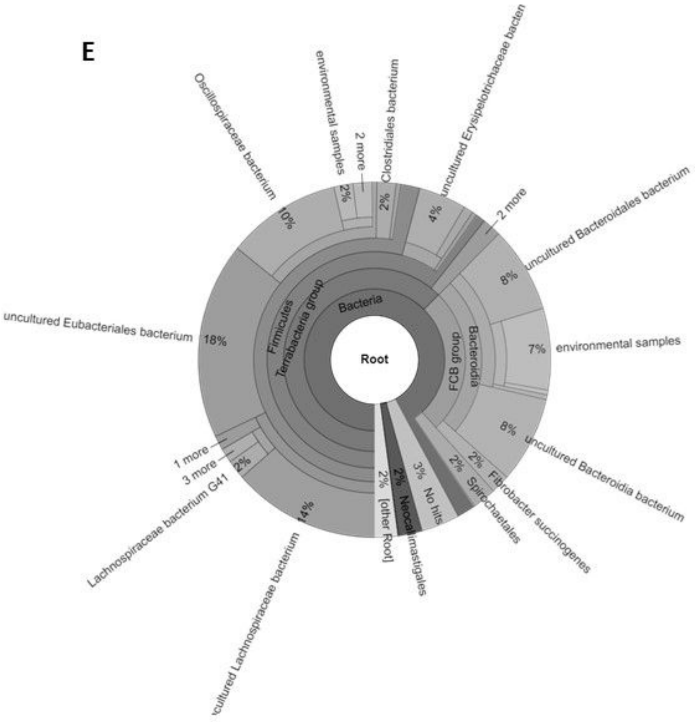
Root
Bacteria
Firmicutes
Terrabacteria group
FCB group
Bacteroidia
Oscillospiraceae bacterium
10%
environmental samples
2%
2 more
Clostridiales bacterium
2%
uncultured Erysipelotrichaceae bacteri
4%
2 more
uncultured Bacteroidales bacterium
8%
environmental samples
7%
uncultured Bacteroidia bacterium
8%
Fibrobacter succinogenes
2%
Spirochaetales
2%
No hits
3%
2%
[other Root]
2%
uncultured Eubacteriales bacterium
18%
1 more
3 more
Lachnospiraceae bacterium G41
2%
uncultured Lachnospiraceae bacterium
14%

F

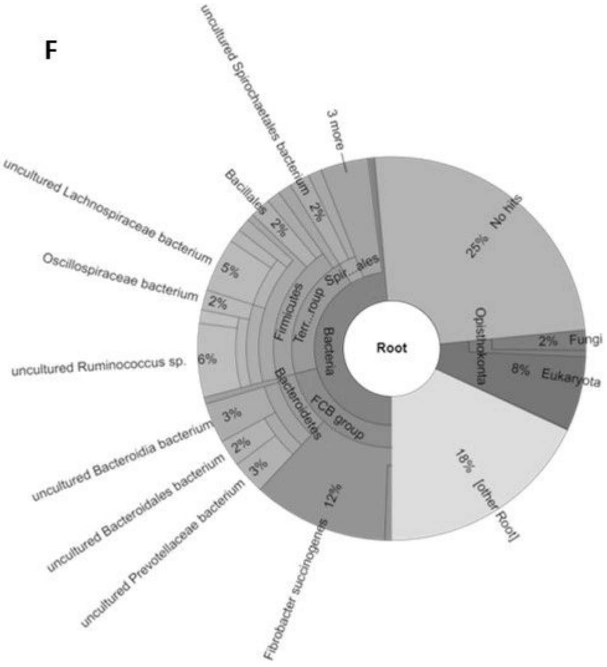
Root
Bacteria
Firmicutes
Terr...roup
Spir...ales
FCB group
Bacteroidetes
uncultured Spirochaetales bacterium
2%
3 more
Bacillales
2%
uncultured Lachnospiraceae bacterium
5%
Oscillospiraceae bacterium
2%
uncultured Ruminococcus sp.
6%
uncultured Bacteroidia bacterium
3%
uncultured Bacteroidales bacterium
2%
uncultured Prevotellaceae bacterium
3%
Fibrobacter succinogenes
12%
No hits
25%
Opisthokonta
Fungi
2%
Eukaryota
8%
[other Root]
18%

Supplementary Figure 3: CAZYME ANNOTATION

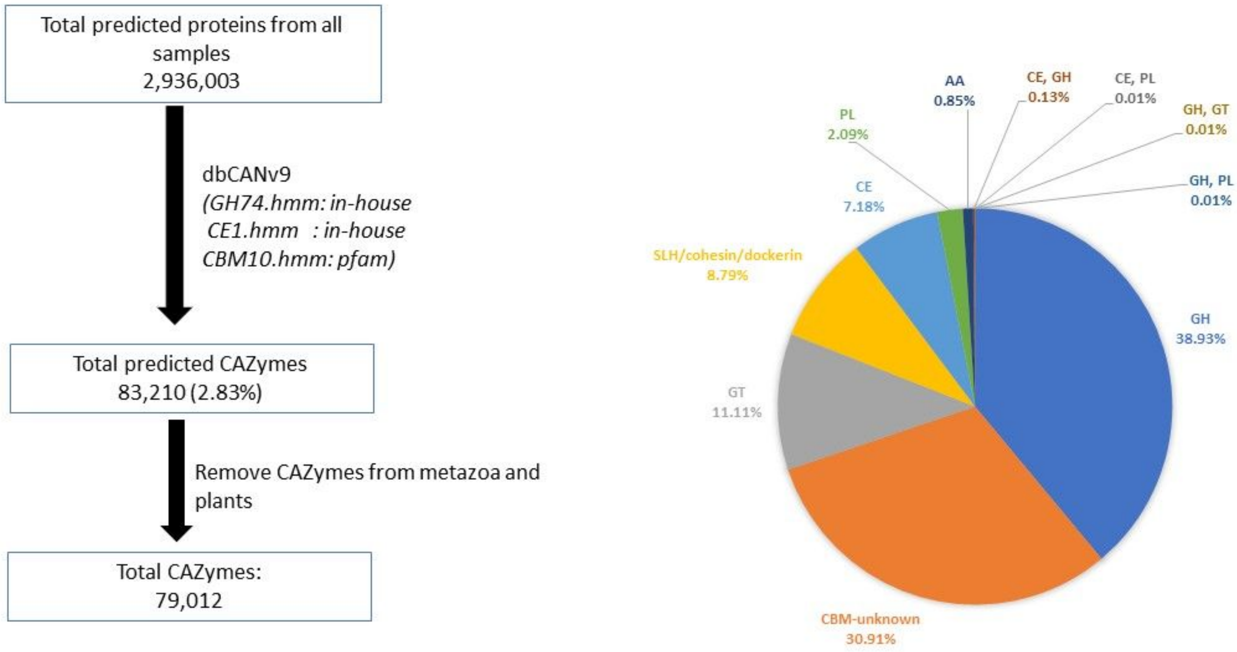

Supplementary Figure 4: Taxonomic distribution of CAZymes

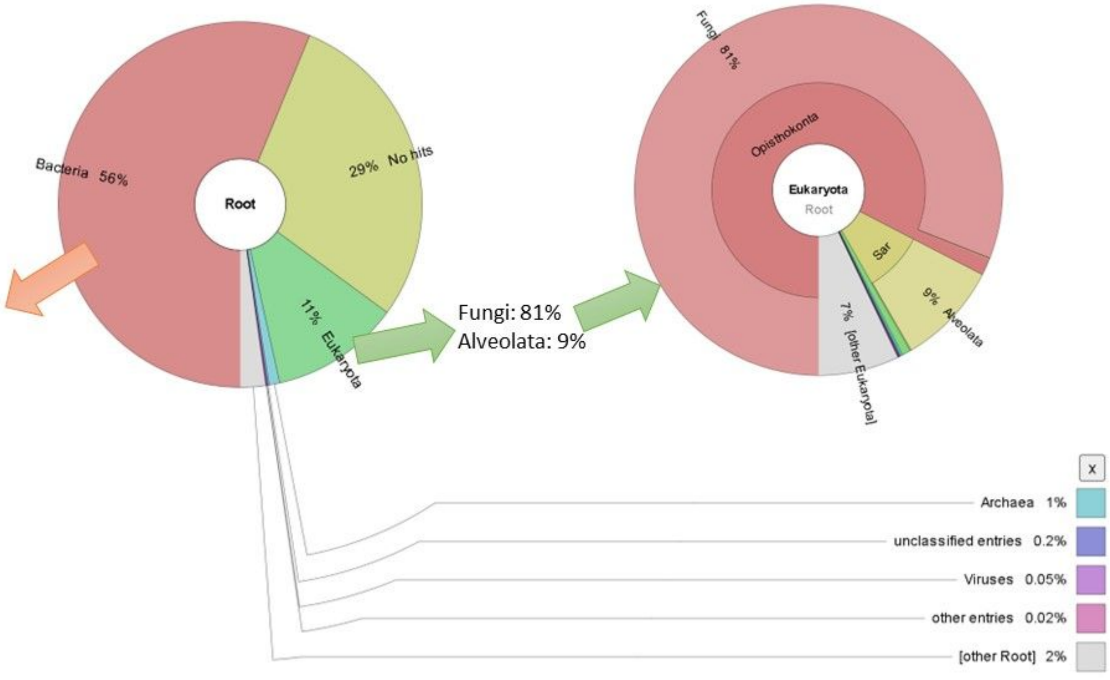

Dominant bacterial phyla:

1. Firmicutes (46%)
2. Bacteroidetes (33%)
3. Fibrobacteres (12%)
4. Spirochaetes (3%)
5. Proteobacteria (2%)

Dominant Genus

1. Ruminococcus (17%)
2. Butyrivibrio (2%)
3. Fibrobacter (12%)
4. Prevotella (2%)

Supplementary Figure 5:Taxonomic distribution of GH proteins

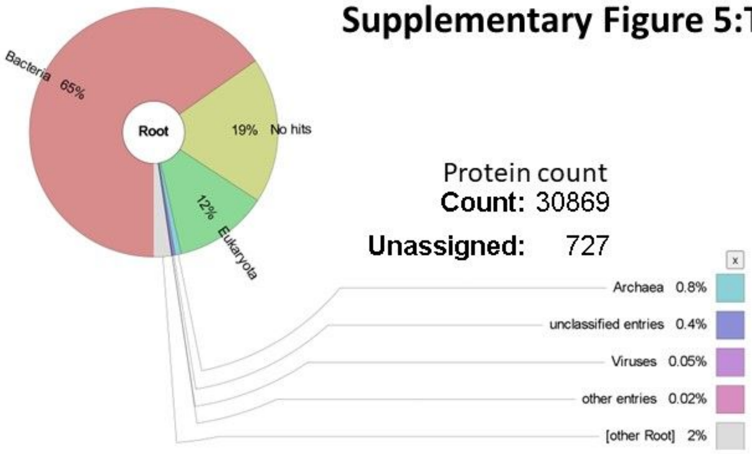

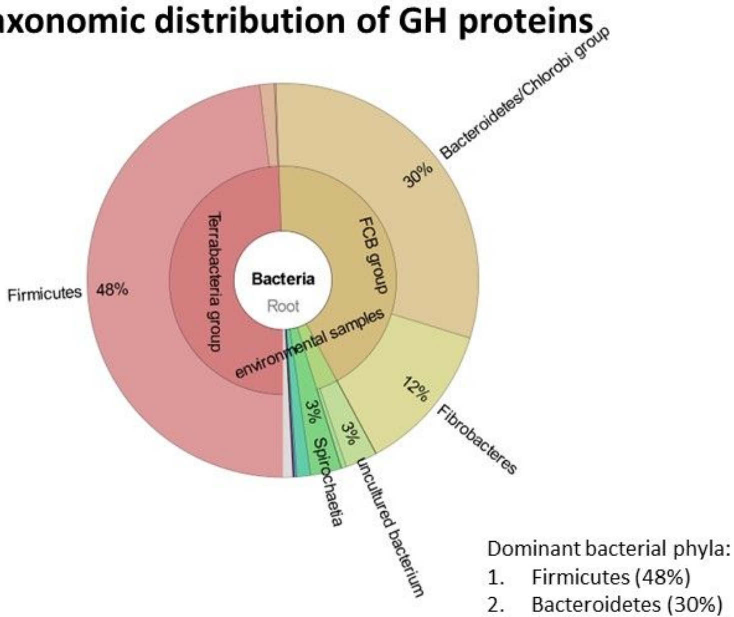

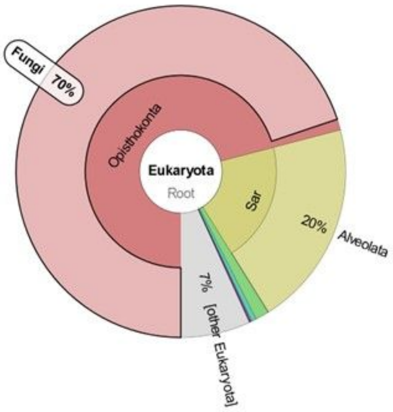

Fungi (mostly Neocallimastigomycetes)

1. Neocallimastix (24%)
2. Caecomycetes (27%)
3. Piromyces (24%)
4. Pecoramyces (10%)
5. Anaeromyces (7%)

Alveolata

1. Ciliophora (99%)

Dominant bacterial phyla:

1. Firmicutes (48%)
2. Bacteroidetes (30%)
3. Fibrobacteres (12%)
4. Spirochaetes (3%)
5. Proteobacteria (1%)

Dominant Genus

1. Ruminococcus (18%)
2. Butyrivibrio (2%)
3. Fibrobacter (12%)
4. Prevotella (2%)

Supplementary Table1 A: RNA sequencing results showing number of reads, contigs and percent mapped-reads from each samples

Samples set	#. of samples	Reads	Contigs	%mapped reads
Cattle-Day0	16	364M	1,642,227	65%
Cattle-Day27	16	383M	1,865,428	65%
Bison	4	107M	467,930	56%

Supplementary Table1 B : Taxonomic mapping of unique contigs

Databases	**Classified contig count**	**Classified contig %**
**Initial RumenDB**<br>- Hungate1000<br>- Neocallimastigomycetes<br>- RefSeq complete bacterial genomes<br>- RefSeq complete archaeal genomes<br>- RefSeq complete protozoan genomes	815,293	29.45%
**913 cow rumen microbial genomes**	810,108	29.26%
Genomic Encyclopedia of Bacteria and Archaea (**GEBA**)	220,078	7.95%
RefSeq complete **fungal** genomes	11,052	0.40%
RefSeq complete **plant** genomes	110,436	3.99%
RefSeq complete **viral** genomes	2070	0.07%
GRCh38 **human** genome	1079	0.04%
**4933 MAGs** from 240 Scottish cattle rumen	1,302,231	47.04%
**NCBI non-redundant nucleotide database**	779,240	28.15%

Total classified contigs (no duplicates) from all 9 DBs: 1,706,811 (61.65%)

Supplementary Table 2: Statistics of Functional annotation, GO and KO annotation.

Functional Description	
Total proteins	2,936,003
Have diamond hits above cut-off (id45q70)	2,293,652 (78%)
Have functional description	2,154,686 (73.39%)
Have description without function information (e.g. hypothetical protein)	3,999,25 (13.62%)
Have description with some functional information	1,754,761 (**59.77%**)

GO Annotation	
Have GO term from at least one GO aspect	1,355,401 (46.1%)
Have Biological Process GO terms	775,757 (26.4%)
Have Molecular Function GO terms	1,104,454 (37.6%)
Have Cellular Component GO terms	708,539 (24.1%)
**KO**	
Assigned to KO groups	1,202,319 (41%)
**EC#**	
Have EC #	43,690 (28%)

Supplementary Table 3: Statistics of Gene oncology and Kegg orthology analysis

	Bison vs Heifer		Day27 vs Day0	
	Protein count	Percent	Protein count	Percent
Total proteins showing significantly differential expression from DESeq2	587301		283855	
Total having function	369163	63%	167557	59%
Total having GO_MF	231073	39%	98536	35%
Total having GO_BP	164740	28%	69719	25%
Total having KO	268509	46%	115162	41%
Total having CAZy domain	21971	4%	10865	4%
